# Subcostal TAPSE: a retrospective analysis of a novel right ventricle function assessment method from the subcostal position in patients with sepsis

**DOI:** 10.1186/s13089-019-0134-7

**Published:** 2019-08-27

**Authors:** Alison B. Main, Rachel Braham, Daniel Campbell, Andrew J. Inglis, Anthony McLean, Sam Orde

**Affiliations:** 1 Alice Springs Hospital, Gap Road, The Gap, NT 0870 Australia; 2 Gold Coast University Hospital, Hospital Boulevard, Southport, QLD 4215 Australia; 3 Royal Darwin Hospital, Rocklands Dr, Tiwi, NT 0810 Australia; 4 St George Hospital, Gray St, Kogarah, NSW 2217 Australia; 5 Nepean Hospital, Derby St, Penrith, NSW 2747 Australia

**Keywords:** Apical four chamber, Right ventricle, Sepsis, Subcostal, Tricuspid annular plane systolic excursion

## Abstract

**Background:**

Tricuspid annular plane systolic excursion (TAPSE) is frequently used as an objective measure of right-ventricular dysfunction. Abnormal TAPSE values are associated with poor prognosis in a number of disease states; however, the measure is not always easy to obtain in the critically ill. The purpose of this study is to assess the feasibility and accuracy of using a subcostal view and TAPSE measurement as a measure of right-ventricular dysfunction. A secondary aim was to perform a pilot study to assess whether right-ventricular dysfunction was associated with adverse outcomes including mortality.

**Results:**

Subcostal TAPSE corresponds well with TAPSE obtained from the apical window at low and moderate TAPSE values (mean difference 1.2 mm (CI 0.04–2.36; 100% data pairs < 3-mm difference for TAPSE < 19 mm; 92% had < 3 mm difference at TAPDE < 24 mm). Subcostal TAPSE is able to accurately discriminate between abnormal and normal TAPSE results (sensitivity 97.8%, specificity 87.5%). There was no association between right-ventricular (RV) dysfunction and 90-day mortality.

**Conclusions:**

Subcostal TAPSE is a feasible and accurate alternative to conventional TAPSE from the apical view in critically ill patients. Further research is required to elucidate the relationship between RV dysfunction and outcomes in sepsis.

## Background

Reversible global myocardial depression is common and occurs early in sepsis and septic shock [1–4]. Echocardiography in the intensive-care unit (ICU) is a crucial tool for the assessment of cardiac function as it is non-invasive, safe, portable, and provides real-time information [5]. However, acquiring images can be challenging in the critically ill, particularly the apical view [6] limiting accurate cardiac assessment [7].

The importance of the role of right ventricle (RV) in the management of the critically ill is being increasingly acknowledged [8–10]. RV dysfunction is common in the ICU, but is often under-recognised in the clinical setting. Dysfunction of the right ventricle in sepsis is progressively more recognised, although this has not been studied to the same extent as the left ventricle [3, 4, 8–10].

RV dysfunction has been associated with increased mortality in sepsis [4, 8, 14], although this has not been uniformly reported [11, 12]. RV dysfunction in sepsis may be characterised by one or more of the following—increased RV dimensions [2, 8, 13], and impaired systolic and/or diastolic function as measured by B- or M-mode or doppler indices (Table 1) [3, 11, 17, 18].

**Table 1:** Commonly used echocardiographic parameters used to assess RV function [18] and evidence of association in sepsis

Echo parameter	Measurement	Abnormal value	Use in assessing RV	Strengths and limitations	Comment	Evidence in sepsis
RV Diameter	Measured at end diastole using focused apical four-chamber view	Basal > 4.2 cm Mid > 3.5 cm	RV dilatation: pressure or volume overload, often precedes RV dysfunction	Easy to perform	RV dilatation occurs in volume and pressure overload	Has not been shown to be predictive of mortality [8]
RV FAC	Endocardial border is traced in end diastole and end systole EDA-ESA/EDA	<35%	Global systolic dysfunction, onerous to perform, difficult to be accurate	“Tedious” to perform [30]	Correlates well with RVEF	Associated with increased mortality [8]
A4C TAPSE	Maximal excursion of tricuspid annulus in longitudinal plane. Measured with M-mode	< 1.6 cm	Utilises longitudinal systolic function as a marker of global RV function	Easy to acquire Low interoperator and intraoperator variability	Prognostic value in multiple disease states including PE, inferior MI, ARDS, critical illness [17, 19, 20]	TAPSE < 2.4 cm predictor of in hospital mortality and longer hospital length of stay [19]
Tissue Doppler TV S′	Apical motion measured using tissue doppler of the tricuspid annulus in systole	< 10 cm/s	Global systolic dysfunction	Easy to measure, reproducible	Correlates well with other measures of RV function	Reduced TV S′ associated with septic shock and increased mortality [3, 9]
E/E′	Ratio of pulsed doppler signal to tissue doppler signal in early diastole	< 6	Elevated in diastolic dysfunction	Easy to perform. Multiple images and measures required	Not valid with significant TR	
IVC	Diameter and respiratory collapse measured from the subcostal window	> 2 cm < 50% collapse = elevated RAP	Measures correlate with estimated RAP	Only possible from one window	> 2 cm < 50% collapse = elevated RAP < 2 cm < 50% collapse 8 mmHg < 2 cm > 50% collapse = RAP 3 mmHg	
RA–RV pressure differential	Tricuspid regurgitant jet commonly used to calculate Peak systolic pulmonary artery pressures, RA pressures estimated from IVC size and variation	> 36 mmHg	Indicates raised pulmonary systolic pressure	Easy to perform. Multiple images and measures required	May be underestimated in right heart systolic dysfunction	
MPI (Tissue Doppler)	Performed using tissue or conventional doppler (IVRT + IVCT)/SEP	> 0.55	Assesses both systolic and diastolic function	Difficult to perform Not dependent on heart rate	May be underestimated in conditions that elevate RA pressures	MPI high in patients with sepsis and septic shock [4, 9]

*FAC* fractional area of change *RVEF* Right ventricle ejection fraction *TAPSE* tricuspid plane annular systolic excursion *MPI* myocardial performance index *IVRT* isovolumetric relaxation time *IVCT* isovolumetric contraction time *SEP* systolic ejection period

**Fig. 1:**
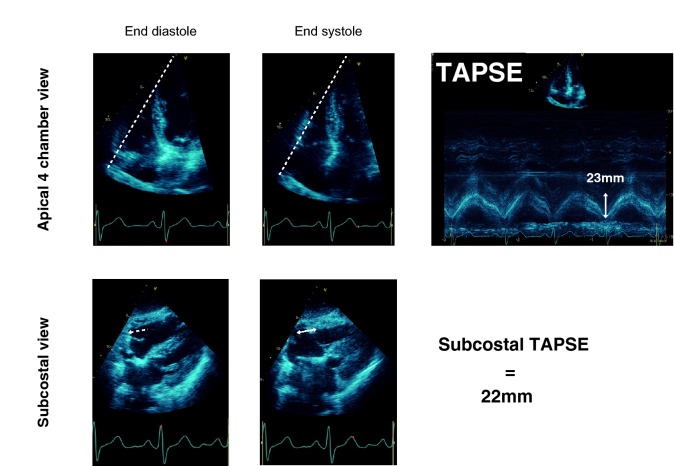
TAPSE measurement. **a** A4C TAPSE: standard method from the A4C window using M-mode. **b** Subcostal TAPSE: measured from subcostal window using B-mode imaging and marker

Right-ventricular contraction mainly occurs in the longitudinal plane. This makes tricuspid annular plane systolic excursion (TAPSE) a suitable method of assessing RV systolic function. TAPSE is usually performed from an apical four-chamber (A4C) window using M-mode across the annulus to evaluate longitudinal contraction (Fig. 1). TAPSE > 16 mm is considered normal; a value < 16 mm indicates reduced RV systolic function. TAPSE closely correlates with RV ejection fraction, and has been shown to be easy to perform, accurate, reproducible with little inter-observer variation [14, 15]. The American Society of Echocardiography currently recommends using TAPSE as one of the tools to assess RV function [16]. It is an independent prognostic marker in critical illness [17] and specific pathologies including pulmonary embolus [18], inferior myocardial infarction [19], and Acute Respiratory Distress Syndrome [20]. However, the utility of TAPSE may be limited by the difficulty in obtaining the A4C echo view. The A4C window often requires repositioning of the patient. In the critically ill, mechanical ventilation, monitoring equipment, wounds, dressings, drains, high body mass index, or chronic lung disease can make imaging from the A4C window challenging (Additional file 1) [7].

The subcostal view is often easier to obtain in the intensive-care population (Additional file 2) [6]. RV function as assessed in the subcostal view has previously been shown to correlate strongly with radionuclide scans [21]. Furthermore, assessment of the tricuspid annulus has been shown to be more frequently possible from the subcostal view [7]. Subcostal TAPSE may, therefore, be an alternative method of assessing RV dysfunction in the critically ill if the traditional A4C view is not available.

The primary aim of this retrospective study was to evaluate the feasibility and accuracy of subcostal TAPSE as a novel method of assessing RV function compared with the conventional TAPSE from the A4C view in intensive-care patients admitted with sepsis.

Sepsis was chosen as a condition that can result in reduced RV function, so that this method could be assessed with both normal and abnormal RV function. Second, this study assessed the association between echocardiographic indicators of RV dysfunction and adverse outcomes, in particular 90-day mortality.

## Methods

### Design, setting, and participants

This is a retrospective observational study performed in Nepean Hospital Intensive Care Unit. Septic patients admitted to the ICU between August 2012 and March 2014 were identified for inclusion in this study.

### Main outcome measures

The primary outcome measure was the comparative accuracy of the TAPSE measurement obtained from the subcostal window compared with the conventional TAPSE measure from the apical four-chamber view. Secondary measures included 90-day mortality.

### Data collection

Patients without echocardiograms, inadequate acoustic windows for analysis, and those who did not meet sepsis criteria were excluded. This study was approved by the Nepean Blue Mountains Local Health District Human Research Ethics Committee (study ref: 14/34) and did not require individual patient consent due to its retrospective and observational nature.

This is intended to serve as a pilot study for future prospective research into the role of RV dysfunction in sepsis outcomes. As such, it was anticipated that approximately 50 participants would be required. The data were obtained from a computerised database.

### Echocardiography

Images were evaluated for conventional measures of RV function: RV dimension, A4C TAPSE, velocity of tricuspid annular systolic motion (TV S′), fractional area change (FAC), tricuspid valve regurgitation peak velocity, estimated RA–RV pressure difference, and IVC diameter in accordance with the latest American Society of Echocardiography guidelines (Table 1) [17]. The subcostal views were given to a different assessor in a blinded fashion, from which the subcostal TAPSE values were measured (S.O.). The subcostal TAPSE was performed using B-mode imaging, in contrast to A4C TAPSE, which utilises M-mode (Fig. 1a). The tricuspid annulus was identified in diastole, and a cursor placed over it. Having evaluated the longitudinal axis, the image was then progressed to systole where a second cursor was placed over the tricuspid annulus. The distance between the two points is the subcostal TAPSE (Fig. 1b).

**Fig. 2:**
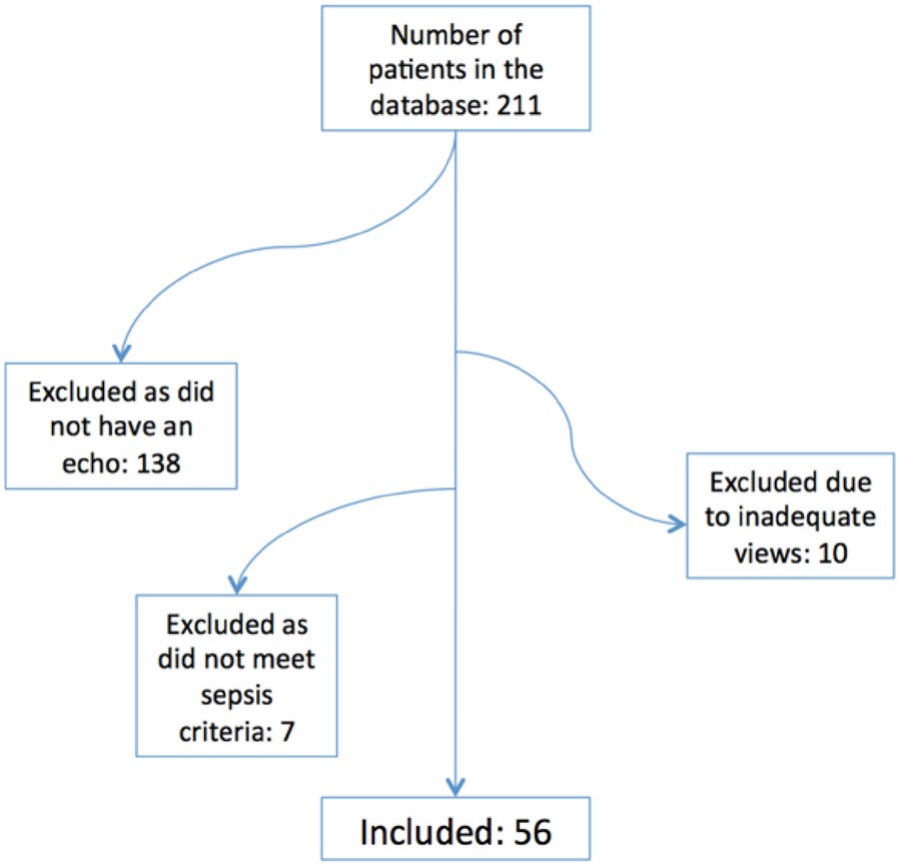
Study design: patients included in study

### Statistical methods

Statistical analysis was performed using JMP 10.0.0 software (SAS Institute Inc., Cary, NC, USA). Continuous data are described using mean ± standard deviation (SD) or median ± interquartile range (IQR) and were analysed between groups using analysis of variance and *t* test. Categorical data are expressed as number of patients and percentage of group and comparisons are made by Pearson’s Chi-squared test or Fisher’s exact test if less than five patients were in a specific group. Probability values are considered two-sided and a p value < 0.05 was considered significant. The correlation between subcostal and A4C TAPSE values was assessed using a Bland–Altman plot and paired *t* test. Categorisation of RV function: “normal” versus “abnormal” function was compared by McNemar’s test, confidence intervals are “exact” Clopper–Pearson confidence intervals. Inter- and intra-observer subcostal TAPSE variability was performed on a random 40% of the study population (23/58 patients) and assessed by Bland–Altman plots as absolute difference and expressed as a percentage of their mean. Mortality was assessed using univariate logistic regression for continuous data and Chi-squared tests for categorical data.

## Results

211 patients were admitted to the intensive-care unit with a diagnosis of sepsis during the study period. 138 patients were excluded as an echo was not performed during their ICU stay, 10 patients were excluded due to inadequate echo views for evaluation (6 inadequate A4C views, 4 subcostal), and 8 patients were excluded as they did not meet criteria [22] for sepsis or septic shock (Fig. 2). A total of 56 patients were included in this study with an average age of 61 (SD 15.3) and mean APACHE III score of 67.5 (SD 27). The characteristics of the study population are described in Table 2.

**Fig. 3:**
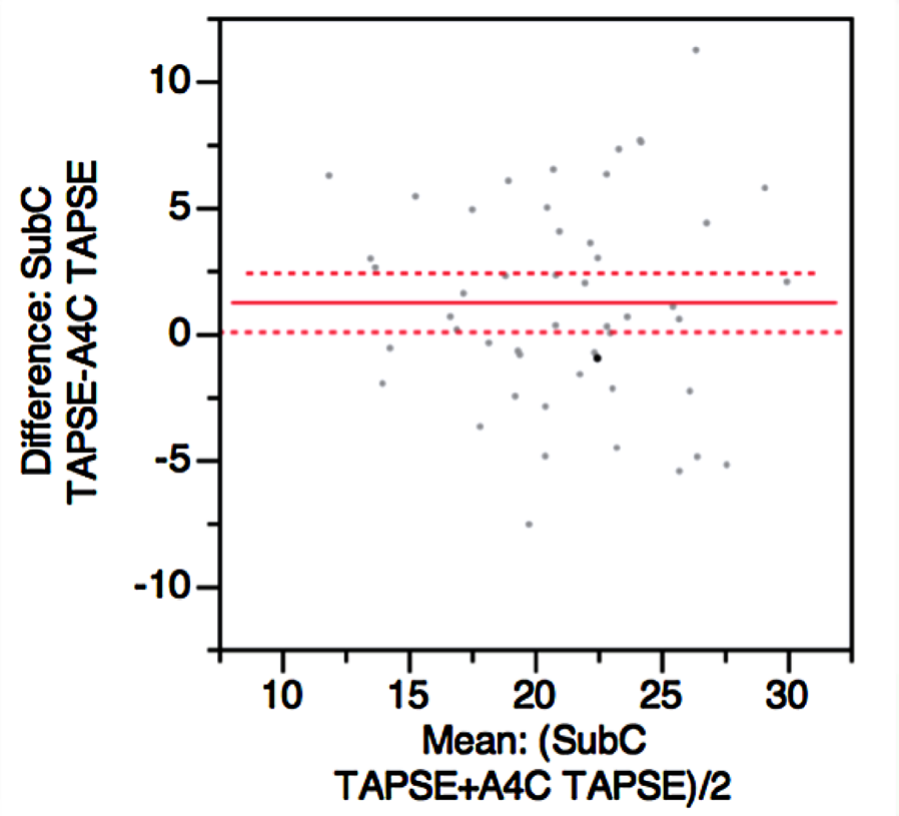
Bland–Altman plot demonstrating mean difference between A4C and Subcostal TAPSE

**Table 2:** Characteristics of study population

Characteristics	90 day mortality		
	Survivors	Non-survivors	*p* value
Age (mean + SD)	59.9 ± 16.1	65.1 ± 12.9	0.2
Female	22 (54.7%)	11 (73.3%)	0.23
Male	19 (46.3%)	4 (26.7%)	
Source of sepsis			
Respiratory	30 (75%)	11 (73.3%)	0.7
Skin	3 (7.5%)	1 (6.7%)	0.9
Urological	4 (10%)	0 (0%)	0.3
Gastrointestinal	1 (2.5%)	1 (6.7%)	0.2
Other	1 (2.5%)	1 (6.7%)	0.47
Unknown	1 (2.5%)	2 (13.33%)	0.17
Apache score mean (SD)	59.8 (20.1)	102.3 (27.6)	0.05

### A4C vs subcostal TAPSE

TAPSE from A4C and subcostal views showed a mean difference of 1.2 mm (CI 0.04–2.36). However, the Bland–Altman plot (Fig. 3) demonstrates most data points outside this confidence interval, with fanning at high TAPSE values.

A difference between subcostal TAPSE and A4C TAPSE of less than 3 mm is present in 92.1% of data pairs when TAPSE < 24 mm; 96.7% of data pairs when TAPSE < 22 mm; and 100% of data pairs when TAPSE < 19 mm. At TAPSE values greater than or equal to 24 mm, 63.6% of values had a difference < 3 mm. Intra-observer variability was good with mean absolute difference (± SD) of 2.3 mm (± 2.7) and expressed as a percentage of the mean 11.4% (± 9). Inter-observer variability was adequate with mean absolute difference of 3.4 mm (± 2.4) and expressed as percentage of the mean 15.8% (± 11).

When subcostal TAPSE is compared with A4C TAPSE as the gold standard, it identifies “Abnormal” TAPSE with a sensitivity of 97.8% (CI 88.2–99.9%) and specificity of 87.5% (47.4–99.7%).

### Right-ventricular dysfunction and 90-day mortality

No correlation was seen between either TAPSE measures of RV dysfunction and 90-day mortality (A4C TAPSE OR = 1.05, CI 0.92–1.20; p = 0.48; subcostal TAPSE OR = 0.98, CI 0.86–1.11 p = 0.74). However, when categorised, “abnormal” TAPSE was associated with higher mortality compared with “normal” TAPSE, although it did not reach statistical significance (57.1% vs 23.9% p = 0.09). Increased RV dimensions at the basal and mid-levels were associated with increased 90-day mortality (basal dimensions OR = 1.16, CI 1.05–1.32, p = 0.004; mid-dimensions OR = 1.12 CI 1.02–1.25, p = 0.012). No other measures of RV dysfunction were associated with increased mortality.

## Discussion

### Apical four-chamber TAPSE versus subcostal TAPSE

In this retrospective, observational study, we demonstrated that subcostal TAPSE correlated well with the conventional A4C TAPSE at low and moderate TAPSE values, with most differences less than 3 mm. The negative prognostic significance of TAPSE values has been reported at values < 18 mm [23], in sepsis, increased mortality has been found at TAPSE values less than 24 mm [17]. It is in this region of interest where subcostal (SC) TAPSE had reasonable accuracy compared with A4C TAPSE. The difference of less than 3 mm is considered to be a clinically acceptable difference. SC TAPSE maintained accuracy when categorising “abnormal” and “normal” TAPSE values.

The mean difference between measures was small; however, most data points fell outside of the confidence intervals, limiting the utility of that measure. There is increased variance at high TAPSE values. The reason for this may be due to translational movement in a hyperdynamic heart or inaccuracies in identifying the axis of contraction with vigorous movement. Although this limits the accuracy of the absolute value of subcostal TAPSE in these circumstances, it does not impede the utility of subcostal TAPSE altogether, as at high values TAPSE is considered to be normal and is unlikely to influence prognostication or interventions and would support an assessment of normal RV function.

Although this data set is small and retrospective, it supports the feasibility of using subcostal TAPSE. Further research is required before implementing its use in clinical practice, particularly ensuring minimal inter-observer variability and discrimination of clinically relevant outcomes.

With greater understanding of the importance of RV dysfunction in critical illness, accurate transthoracic echo assessment of the RV is a crucial tool in managing intensive-care patients. Subjective assessment of RV function has been shown to only have fair reliability when performed by ICU specialists adequately trained in echo, but only for initial exclusion of significant RV pathology. Quantification of RV function is important to ensure accurate estimation of performance [24].

TAPSE is a commonly used tool in cardiac function analysis; however, its utility is limited in the critically ill due to difficulty obtaining A4C views. Inability to obtain TAPSE has been reported to range from 6 to 25% of cases [25–27]. Other measures of RV function such as Fractional Area of Change have been described as “tedious” with TAPSE comparably easier [28]. Subcostal views have previously been shown to be comparable to A4C views in the assessment of RV function [21]. Most TAPSE measurements take less than 30 s to perform. The relative ease of obtaining images from the subcostal window makes this an attractive alternative in the critically ill.

The use of the subcostal window to assess the RV has been increasingly considered, although with varied success [7, 29]. Subcostal echocardiographic assessment of tricuspid annular kick (SEATAK) has been purported as an alternative to TAPSE in critically ill patients [7]. SEATAK utilises the subcostal short-axis view; using M-mode, the cursor placed over the tricuspid annulus and measured in a similar way to A4C TAPSE measurements. This study confirmed the feasibility of using the subcostal view; however, the angle of M-mode may underestimate RV function. Subcostal TAPSE has the advantage of identifying and measuring in the longitudinal axis along which the RV contracts.

### RV function and mortality

RV dysfunction in critical illness and sepsis, in particular, has been associated with increased mortality, [11, 30]; however, the association between specific echocardiographic measures and outcomes have not been consistently reported [3, 4, 9, 13, 31].

TAPSE has been demonstrated to accurately measure RV function and predict mortality in a variety of conditions [14, 18–20, 25]. Reduced TAPSE has been associated with increased mortality and longer hospital length of stay [17], although this finding has not been replicated [4]. In this study, there was no association between TAPSE and 90-day mortality. There are a number of possible reasons for this: the numbers in this study are small and may not suffice to show any difference if present; there are a complex variety of factors that result in adverse outcomes in sepsis; TAPSE alone may not be a prognostically significant marker. In addition, the timing of the echocardiogram was not controlled for. As such, the findings may be affected by fluid resuscitation, inotropic and ventilator support or the natural course of the illness. Further investigation is required to assess the utility of subcostal TAPSE in assessing RV function in sepsis and its role in prognostication. In particular, standardisation of timing of the echocardiogram (such as when inotropic or vasoactive medications are commenced, or serially at predetermined times during the ICU stay) may aid in identifying an association with RV dysfunction and outcomes. Based on these data, a study to detect an association between TAPSE and mortality would require a sample size of at least 638 utilising a 1 mm difference in TAPSE with a standard deviation of 4.5, an alpha of 0.05 and 80% power.

Interestingly, there was an association between increased RV diameter and mortality in this population. The reason for this association is not clear; however, increased end-diastolic volume (EDV) has previously reported in sepsis, although it has not been associated with an increase in mortality [2, 8, 32]. Increase in RV diameter occurs in both pressure and volume overload [33–35]. We hypothesise that EDV may be a surrogate for fluid overload. As excess fluid has been associated with increased mortality in sepsis [36–38], this provides a possible link between the RV measurements and mortality outcomes. Other causes of right-ventricular dilatation, such as pulmonary hypertension associated with sepsis and high intrathoracic pressures with positive pressure ventilation, may contribute to RV dilatation, but are beyond the scope of this study. Further investigation is required to evaluate the relationship, if any, between fluid status, RV dimensions, and mortality.

### Strengths and limitations

The main strengths of this study are that it supports the feasibility of a novel echo technique and it generates further hypotheses regarding the role of the RV in sepsis and provides direction for further research in this area. This study is limited by its retrospective nature and small numbers. A number of patients were excluded, because there were no echocardiograms performed. In addition, the timing of the echo was not controlled for, which may influence the accuracy of using TAPSE as a prognostic tool. The exclusion of patients without echocardiograms would not impact our primary research aim, and the overall numbers excluded for inadequate subcostal views were small. Exclusion of patients may have reduced overall numbers, so that finding associations, if any exist, between adverse outcomes and RV dysfunction is less likely. Reassuringly, the association between A4C and subcostal TAPSE that has been found is likely to be robust, within the limits of a small study, as the patient characteristics are similar to patient groups in other published sepsis trials [38].

Another limitation of this trial was that one clinician performed all subcostal TAPSE measurements. Despite this, inter-observer variation was adequate, and intra-observer variation was good in a subset of measures. This method needs to be further assessed before general use ensuring accuracy persists with multiple clinicians.

## Conclusion

Subcostal TAPSE correlates well with the conventional A4C TAPSE at low and moderate TAPSE values and categorises TAPSE into “normal” and “abnormal” with good accuracy. SC TAPSE may be a surrogate method for RV analysis in the critically ill where apical views are challenging. Subcostal TAPSE is quick, easy, and provides significant information about RV function. With growing use of point of care ultrasound and focused echo in critical care, subcostal TAPSE could quickly and easily be added to the examination; however, confirmation of minimal inter-observer variability is required first. This study suggests that increased EDV may be associated with increased mortality. The importance of RV dysfunction in sepsis and its sequelae require further exploration.

## Supplementary information

**Additional file 1: Video S1.** Apical four-chamber image was challenging to obtain and the tricuspid annulus is less well seen.

**Additional file 2: Video S2.** Subcostal image from the same patient, the tricuspid annulus has better definition.

## Data Availability

The data sets generated and analysed during this study are available from the corresponding author on reasonable request.

## Abbreviations

A4C: apical four chamber
EDV: end diastolic volume
eGFR: estimated glomerular filtration rate
FAC: fractional area change
ICU: intensive-care unit
RV: right ventricle
SC: subcostal
SEATAK: subcostal echocardiographic assessment of tricuspid annular kick
TAPSE: tricuspid annular plane systolic excursion
TV S′: tricuspid annular systolic motion velocity
